# Effect of *n*- and *p*-Doping on Vacancy Formation in Cationic and Anionic Sublattices of (In,Al)As/AlAs and Al(Sb,As)/AlAs Heterostructures

**DOI:** 10.3390/nano13142136

**Published:** 2023-07-23

**Authors:** Timur S. Shamirzaev, Victor V. Atuchin

**Affiliations:** 1 Laboratory of Physics and Technology of Heterostructures, Institute of Semiconductor Physics, SB RAS, Novosibirsk 630090, Russia; tim@isp.nsc.ru; 2 Department of Physics, Novosibirsk State University, Novosibirsk 630090, Russia; 3 Laboratory of Optical Materials and Structures, Institute of Semiconductor Physics, SB RAS, Novosibirsk 630090, Russia; 4 Department of Applied Physics, Novosibirsk State University, Novosibirsk 630090, Russia; 5 Research and Development Department, Kemerovo State University, Kemerovo 650000, Russia; 6 Department of Industrial Machinery Design, Novosibirsk State Technical University, Novosibirsk 630073, Russia; 7 R&D Center “Advanced Electronic Technologies”, Tomsk State University, Tomsk 634034, Russia

**Keywords:** vacancy generation, recombination, semiconductor heterostructure, diffusion

## Abstract

The vacancy generation dynamics in doped semiconductor heterostructures with quantum dots (QD) formed in the cationic and anionic sublattices of AlAs is studied. We demonstrate experimentally that the vacancy-mediated high temperature diffusion is enhanced (suppressed) in n- and p-doped heterostructures with QDs formed in the cationic sublattice, while the opposite behavior occurs in the heterostructures with QDs formed in the anionic sublattice. A model describing the doping effect on the vacancy generation dynamics is developed. The effect of nonuniform charge carrier spatial distribution arisen in heterostructures at high temperatures on the vacancy generation and diffusion is revealed.

## 1. Introduction

The carrier confinement obtained in semiconductor quantum wells (QWs) and quantum dots (QDs) attracts research attention due to their unique electronic and optical properties [1,2,3,4,5,6,7,8,9,10,11,12,13]. In heterostructures with quantum dots and quantum wells at high temperature, the vacancy formation leads to the material intermixing via a vacancy-mediated diffusion [14,15]. The intermixing permits modifying the energy structure [14,15,16,17,18], controls hyperfine interaction [19], adjusts exciton lifetime [17,20], and reduces strain gradients [21,22] in low dimensional structures.

Vacancies in semiconductors can be electrically charged [23,24,25]. Therefore, a strong effect of the charge-carrier concentration on the vacancy generation occurs [15,23,26,27,28,29]. The vacancy generation dependence on the carrier concentration was studied well in bulk crystals [23,26,27,30,31]. In contradistinction to bulk crystals with uniform charge carrier distribution, a redistribution of charged carriers between different layers occurs in heterostructures. We have shown recently that the redistribution of thermally generated equilibrium electrons in undoped heterostructures can strongly change the distribution of vacancy generation in real space and vacancy-mediated diffusion [16].

In this paper, we investigate the doping effect on the charged vacancy formation in cationic and anionic sublattices of bulk AlAs and the vacancy-stimulated diffusion in heterostructures with InAlAs/AlAs and AlSbAs/AlAs QDs. It is experimentally shown that the material intermixing degree upon the annealing at a fixed temperature in the cationic sublattice is the lowest in a *p*-doped heterostructure, increasing in an undoped one, and becomes the highest in an *n*-doped heterostructure. For the anionic sublattice, the behavior is the opposite: the lowest intermixing degree takes place in an *n*-doped heterostructure and the highest one in a *p*-doped one. The experimental observation is explained in the framework of a theoretical model, taking into account the strong dependence of the charged vacancy formation rate on the charge carrier density in the bulk material and nonuniform spatial distribution of charged carriers within a heterostructure.

The paper has the following structure. In Section 2, the heterostructures and the experimental techniques are described. In Section 3, one can see the experimental data on the vacancy-mediated material intermixing in undoped as well as *n*- and *p*-doped heterostructures with QDs, formed in cationic (InAs) and anionic (AlSb) sublattices of AlAs as the functions of temperature and doping level. The theoretical model describing the negatively and positively charged vacancy formation dynamics in bulk AlAs, as well as AlAs-based heterostructures with QDs, and the model calculations of the vacancy distribution and vacancy-mediated intermixing are presented in Section 4.

## 2. Experiments

Heterostructures with InAs (AlSb) QDs in an AlAs matrix were grown by molecular beam epitaxy on semi-insulating (001)-oriented GaAs substrates in a Riber Compact system. The samples consisted of one layer of the QDs sandwiched between AlAs layers grown on top of a 400 nm thick GaAs buffer layer. The lower AlAs layer with 50-nm thickness was grown in all structures at the temperature of 620 °C. Then the growth was interrupted, and the substrate temperature was decreased down to 480 °C under the As flux during cooling. The InAs (AlSb) QDs were formed at 480 °C. The upper 80-nm thick AlAs layer was grown at the same substrate temperature as the corresponding InAs or AlSb layers. A 20-nm thick cap GaAs layer was grown on the top of the AlAs layer for protection. Further details for the epitaxial growth in the AlAs matrix are given in [32,33,34,35].

From the growth conditions and model calculations, we can conclude that the average composition for (In,Al)As/AlAs QDs is about In_0.75_Al_0.25_As [32] and, for Al(Sb,As)/AlAs QDs, it is about AlSb_0.3_As_0.7_ [33]. The size and density of the lens-shaped QDs have been recently determined by transmission electron microscopy, yielding the average diameter (*D*_av_) of 12 nm (the larger *D*_L_ is 16 nm and the smaller diameter *D*_S_ (8 nm) in half-widths of the QD size distribution) and a density of about 2 × 10^10^ dots per cm^2^ for (In,Al)As/AlAs QDs [32], and the average diameter of 20 nm (the larger *D*_L_ is 25 nm and the smaller *D*_S_ (11 nm) in half-widths of the QD size distribution) and a density of about 5 × 10^10^ dots per cm^2^ for Al(Sb,As)/AlAs QDs [33]. The relatively low QD density prevents the carrier redistribution between the QDs [36,37].

**Figure 1:**
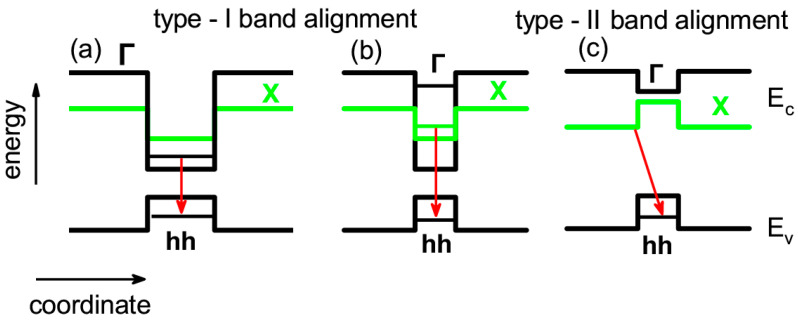
Schematic band diagrams of heterostructures. (**a**) Type-I direct band gap and (**b**) type-I indirect band gap, in InAlAs/AlAs QDs with large and small size; (**c**) indirect band gap with type-II in AlSbAs/AlAs QDs. Red arrows mark optical transitions of the exciton to the system ground state.

The QDs formed in cationic and anionic sublattices of AlAs have different energy spectra. In the case of (In,Al)As/AlAs QDs, the dispersion in dot size, shape, and composition within the ensemble leads to the formation of two configurations, shown in Figure 1a,b. The electron ground state shifts from the Г- to the X-valley with the decreasing dot diameter, while the heavy-hole (hh) ground state remains at the Г point. This corresponds to a change from a direct to an indirect bandgap in the momentum space, while the band alignment of type-I is preserved; that is, in both cases, an electron and a hole are spatially confined within the (In,Al)As QDs [38,39]. The Al(Sb,As)/AlAs QDs have a band alignment of type-II with the lowest conduction-band states at the X_xy_ minima of the AlAs conduction band and the heavy-hole (hh) ground state at the Г point, which leads to the band structure scheme in Figure 1c [33].

**Figure 2:**
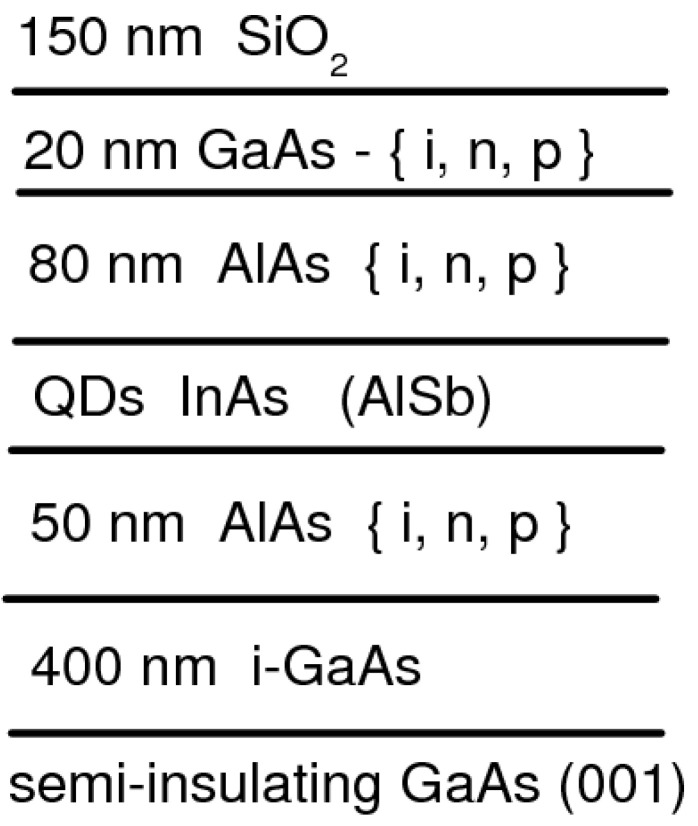
Scheme of the doping design in the heterostructures with QDs.

The AlAs barriers in the studied structures were undoped or doped with donors (silicon) to about 3 × 10^18^ cm^−3^, or acceptors (beryllium) to about 5 × 10^18^ cm^−3^. The doping designs in the heterostructures with QDs are shown in Figure 2. The samples were annealed at different temperatures (*T*_A_) in the range from 550 to 770 °C in a hydrogen flow for 10 minutes. To prevent the surface decomposition during the annealing, the samples were protected by a 150 nm thick SiO_2_ layer. The degree of material intermixing upon annealing was estimated from the emission blue-shift in the photoluminescence (PL) spectra of the heterostructures that occurs due to a decrease in the confining potential as a result of a change in the QD size and composition [22]. The samples were placed in a cryostat and the temperature for all experiments was fixed at *T* = 10 K. A semiconductor laser (3.07 eV) with an excitation power density of 25 W/cm^2^ was used for the PL excitation. The emitted light was dispersed by a 0.5-m monochromator and detected by a cooled CCD.

## 3. Experimental Results

**Figure 3:**
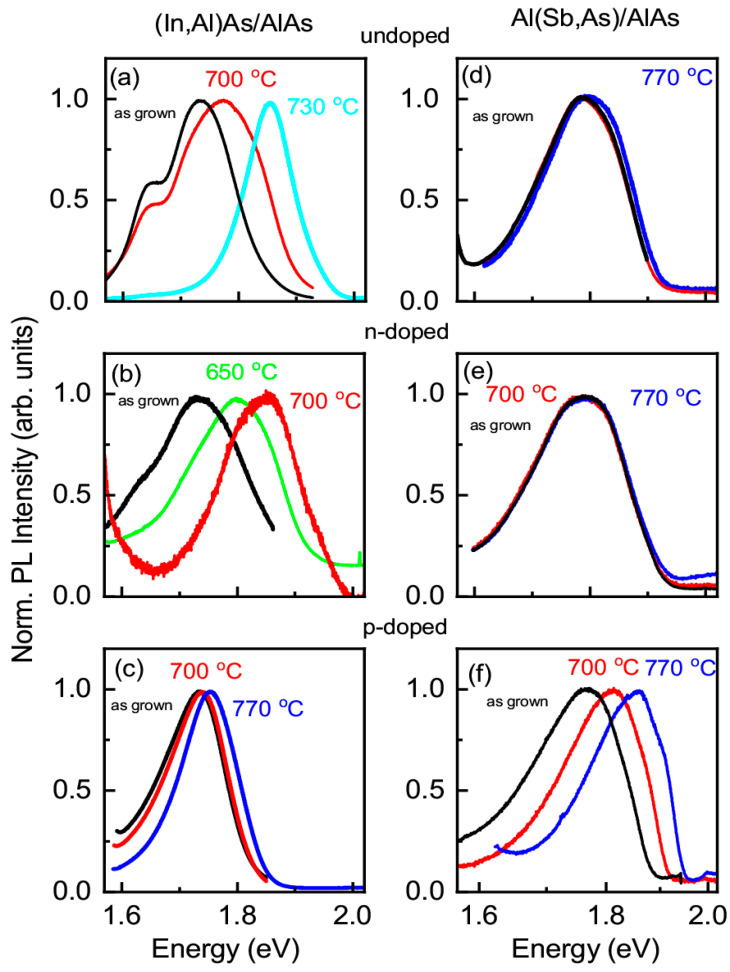
PL spectra measured at 10 K of (**a**–**c**) (In,Al)As/AlAs QDs and with (**d**–**f**) Al(Sb,As)/AlAs QDs annealed at different temperatures: as-grown (black), 650 °C (green), 700 °C (red), 730 °C (cyan), and 770 °C (blue); (**a**,**d**) undoped, (**b**,**e**) *n*-doped (with donor concentration 3 × 10^18^ cm^−3^), (**c**,**f**) *p*-doped (with acceptor concentration 5 × 10^18^ cm^−3^) heterostructures.

The PL spectra of undoped heterostructures with (In,Al)As/AlAs and Al(Sb,As)/AlAs QDs annealed at different temperatures are shown in Figure 3a,d, respectively. The spectrum of the unannealed (as-grown) structure (black line) has its maximum *PL*_max_ = 1.72 eV (1.78 eV) for the emission in indirect bandgap QDs (In,Al)As with the band diagram shown in Figure 1b (band diagram of Al(Sb,As) QDs shown in Figure 1c.) The large emission band width is due to the dispersion of the QD parameters since the exciton energy depends on the QD size, shape, and composition [32,33]. The low energy shoulder in the spectra of (In,Al)As/AlAs QDs is a contribution of direct bandgap QDs (see Figure 1a) as it was shown in [32,40]. The spectra of the as-grown *n*-doped (with donor concentration 5 × 10^18^ cm^−3^) and *p*-doped (with acceptor concentration 5 × 10^18^ cm^−3^) heterostructures have a similar shape (see Figure 3). A 10 min annealing at *T*_A_ = 550 °C and below does not affect the shape of the photoluminescence spectra for all structures. With further increases in the annealing temperature, the PL band monotonically shifts to the high-energy region of the spectrum (demonstrates the blue-shift). One can see that the annealing temperature corresponding to the onset of the PL band blue-shift and to the blue-shift magnitude at a specific temperature depends on the doping type and level. The PL band shape reflects the QDs’ size distribution. Since the PL band shape is changed with annealing mainly due to a stronger shift of the low energy side of the PL band, we can conclude that interdiffusion is more pronounced in large-size QDs than that in small-size QDs.

**Figure 4:**
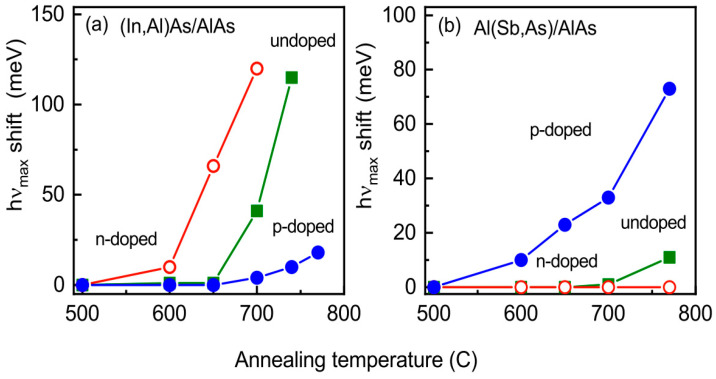
Shift of the PL band maximum position in the spectra with (**a**) (In,Al)As/AlAs QDs and (**b**) Al(Sb,As)/AlAs QDs with different doping types and levels depending on the annealing temperature. Undoped (olive), *n*-doped (with donor concentration 3 × 10^18^ cm^−3^) (red), and *p*-doped (with acceptor concentration 5 × 10^18^ cm^−3^) (blue).

In the heterostructure with *n*-doped (In,Al)As/AlAs QDs, the PL band blue-shift is observed already at *T*_A_ = 600 °C, and such shift is increased strongly at higher temperatures, as it is seen in Figure 3b. For the undoped and *p*-doped heterostructures with (In,Al)As/AlAs QDs, the blue-shift takes place above *T*_A_ = 650 °C (see Figure 3a,c), and, in the *p*-doped heterostructure, this shift, at a fixed temperature in the range from 650 °C up to 770 °C, is noticeably smaller than that in the undoped one. For heterostructures with Al(Sb,As)/AlAs QDs, the blue-shift is observed already at *T*_A_ = 600 °C in the case of *p*-doping, while, in the case of *n*-doping, we do not have any blue-shift up to 770 °C (see Figure 3d,f). The shifts of the PL band maximum position relative to *hν*_max_ of the as-grown structures as a function of annealing temperature for the heterostructures with QDs formed in the cationic and anionic sublattices are collected in Figure 4a,b, respectively.

Let us summarize the most important experimental findings:

(1) The intermixing of materials, that leads to a high-energy shift of the PL band due to the QD atom diffusion into the AlAs matrix during high-temperature annealing, depends strongly on the level and type of doping.
(2) For (In,Al)As quantum dots forming in the AlAs cationic sublattice, this blue-shift under the same annealing conditions is the smallest in the *p*-doped heterostructure, increases in the undoped one, and becomes the largest in the *n*-doped heterostructure.
(3) The Al(Sb,As)/AlAs quantum dots forming in the AlAs anionic sublattice demonstrate the directly opposite behavior: the smallest blue-shift occurs for the *n*-doped heterostructure and the largest one for the *p*-doped heterostructure.

## 4. Discussion

The material intermixing in the studied heterostructures occurs through the vacancy formation in the corresponding AlAs sublattice. Therefore, the material intermixing degree during annealing is determined by the vacancy formation rate [41]. In the next subsection, we extend our model, developed in [16] for the vacancy formation in the cationic sublattice of neutral crystals, and consider the vacancy formation dynamics in the cationic and anionic sublattices of AlAs depending on the doping level.

### 4.1. Vacancy Formation Dynamics

Vacancies can be formed in different mechanisms. The main paths are a generation at the surface (Schottky defect formation) and in the volume (Frenkel pair formation). In the first case, to consider the vacancy formation, it is necessary to take into account the surface structure [42,43,44,45,46] and the free surface exchange by atoms and molecules with the environment [28,47,48,49]. Frenkel pair formation is considered an unlikely source of vacancies for most semiconductor materials due to the high formation energy. However, here we focus on the processes occurring deep in the material volume only. Therefore, we neglect the defects formation and recombination at the surface (Schottky defects), as well as the defects diffusion from and to the surface. The vacancy density in our study is ruled by the temperature-activated generation of Frenkel pairs (interstitial atom and vacancy). We also do not take into account the complex defects creation, such as bi-vacancies and vacancy-point defect complexes. In a uniform bulk crystal, the vacancy concentration (*N_V_*) dynamics are described by the equation:

(1) $$\frac{\partial N_{V}(t)}{\partial t}=G(t)-R(t),$$

where *G* is a vacancy formation rate and *R* is a vacancy recombination rate. The vacancy formation rate is the Arrhenius function $A\cdot \exp\left(-H_{A}/kT\right)$ with pre-exponential factor *A*, activation enthalpy *H_A_*, and *k* is the Boltzmann constant. The pre-exponential factor can be written as:

(2) $$A=\gamma_{c}N_{AP}N_{IP}\nu \exp\left[\frac{S_{f}+S_{m}}{k}\right],$$

where *N_AP_* is the number of atoms that can go to an interstitial place with a vacancy formation, *N_IP_* is the number of interstitial places near *N*_AP_ atoms (in a uniform bulk crystal *N_AP_* = *N_IP_* = *N*, where *N* is the atom density in an appropriate sublattice), *ν* is the Debye frequency, *S_f_* and *S_m_* are formation and migration entropies, and *γ_c_* is a coefficient that depends on the interaction mechanism between a vacancy and an interstitial atom. We have shown recently that one can use $\gamma_{c}=a^{3}$, where a is a lattice constant [16]. The activation enthalpy of the vacancy formation is $H_{A}^{}=H_{f}^{}+H_{m}^{}$, where $H_{f}^{}$ is a formation enthalpy and $H_{m}^{}$ is a migration enthalpy [26,30]. Note that, typically, the interstitial migration has lower migration barriers, compared to vacancies [50]. Therefore, we relate here $H_{m}^{}$ to the interstitial atom migration. The vacancies recombination rate can be written as:

(3) $$R_{}^{}=a^{3}N_{V}^{}N_{I}^{}\nu \exp\left[\frac{S_{m}}{k}\right]\exp\left[-\frac{H_{m}^{}}{kT}\right],$$

with an interstitial atom and vacancies concentration $N_{I}$ and $N_{V}^{}$, respectively. One can write the neutral vacancy formation and recombination rates in the following manner:

(4a) $$G=N^{2}G^{0},\;G^{0}=a^{3}\nu \exp\left[\frac{S_{f}+S_{m}}{kT}\right]\exp\left[-\frac{H_{f}^{0}+H_{m}^{0}}{kT}\right]$$

(4b) $$R_{}^{}=N_{V}^{}N_{I}^{}R^{0},\;R_{}^{0}=a^{3}\nu \exp\left[\frac{S_{m}}{k}\right]\exp\left[-\frac{H_{m}^{}}{kT}\right],$$

where $H_{f}^{0}$ and $H_{m}^{0}$ are neutral vacancy formation and migration enthalpies, respectively.

The solution of Equation (1), taking into account Expressions (4a) and (4b), gives, in thermodynamic equilibrium, a well-known expression for the equilibrium concentration for neutral vacancies formed by the Frenkel mechanism, which does not depend on the state of the crystal electronic subsystem:

(5) $$N_{V}^{0}=N\exp\left[\frac{S_{f}^{}}{2k}\right]\exp\left[-\frac{H_{f}^{0}}{2kT}\right].\;$$

The generation of neutral vacancies induces a change in the atomic subsystem of a crystal only, and doping does not affect this process. Here, we will consider the charged vacancy formation dynamics in doped heterostructures. To simplify it, we will use the wide-gap crystal approximation, in which the intrinsic electron and hole concentrations are much smaller than the dopant concentration. In addition, we will assume, for simplicity, that the vacancy can be neutral or have different charge states, while the interstitial atom is neutral. The recombination of Frenkel pair occurs only when the interstitial atom is located in the nearest internode to the vacancy (short-range interaction limit).

**Figure 5:**
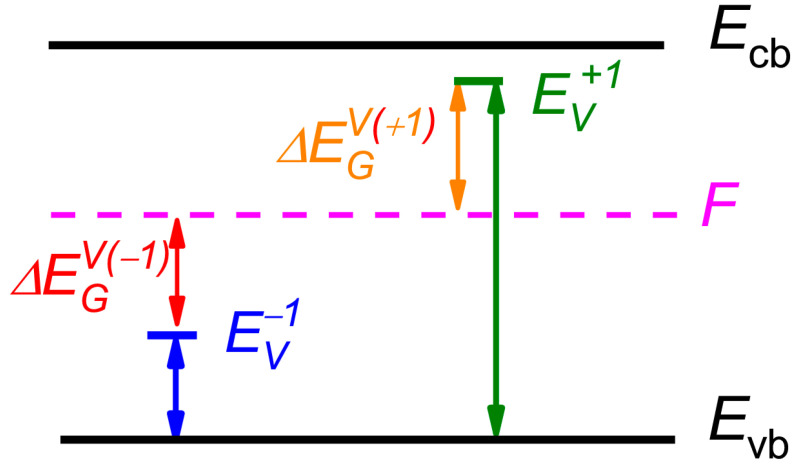
Band diagram for charged vacancies. Vertical arrows: olive and blue—positive and negative ionization energy of a charged vacancy, red and orange—energy gap between the vacancy ionization energy and Fermi energy (dashed magenta). Zero energy level is the valence band top.

The charged vacancy activation enthalpy is $H_{A}^{j}=H_{A}^{0}+\Delta E_{G}^{V(j)}$. The index *j* shows the charge state of vacancy. For the negatively charged vacancy, the term $\Delta E_{G}^{V(-j)}$ equals $\sum_{i=1}^{j}\left(E_{V}^{i}-F\right)$, where *F* is the Fermi energy and $E_{V}^{i}$ is the -*i* charged vacancy ionization energy [23,26,29], while for the positively charged vacancy $\Delta E_{G}^{V(+j)}=\sum_{l=1}^{j}\left(F-E_{V}^{l}\right)$, where $E_{V}^{l}$ is the +*l* charged vacancy ionization energy as it is shown schematically in Figure 5 for single-charged vacancies. Here and below, all energies are measured relative to the top of the valence band (*E*_vb_).

For the negatively charged vacancy that captures *j* electrons at the formation, the pre-exponential factor is corrected to a probability of these electrons to be at the spatial point of the vacancy formation, since an electron’s probability to be at the crystal cell, where the vacancy is created, equals the ratio of free electron concentration (*n*) to the conduction band density of states (*N_ce_*) [51]. In a uniform bulk crystal, one has $N_{AP}^{}=N_{IP}^{}=N{\left(n/N_{ce}^{}\right)}^{j}$. For a positively charged vacancy that emits *l* electrons at generation, on the contrary, the pre-exponential factor is corrected to a probability to have a free place for the emitted electrons at the spatial point of the vacancy formation $N_{AP}^{}=N_{IP}^{}=N{\left(p/N_{vh}^{}\right)}^{l}$, where *p* is a free hole concentration and *N_vh_* is a valence band density of states [51].

For the case of a non-degenerate semiconductor, when $E_{cb}-F\leq kT$ ($F-E_{vb}\leq kT$), where *E*_cb_ is the conduction band edge energy, there is a relation $n=N_{ce}\exp[(F-E_{cb}^{})/kT]$ ($p=N_{vh}\exp[(E_{vb}-F)/kT]$). Therefore, the product of the cells number in which a *j*(*l*)-charged vacancy can be formed and the correspondent interstitials number is $N_{AP}\;\times \;N_{IP}=N^{2}{\left(n/N_{ce}\right)}^{2j}=N^{2}\exp[2j(F-E_{cb})/kT]$ ($N^{2}{\left(p/N_{vh}\right)}^{2l}=N^{2}\exp[-2lF/kT]$), and one can write the vacancy formation rate for a negatively (positively) charged vacancy as:

(6a) $$G_{}^{-j}=a^{3}\nu \;N_{}^{2}\exp\left[2j\frac{F-E_{cb}}{kT}\right]\exp\left[\frac{S_{f}+S_{m}}{k}\right]\exp\left[-\frac{H_{A}^{0}+\sum_{i=0}^{j}E_{V}^{i}-jF}{kT}\right],$$

(6b) $$G_{}^{+l}=a^{3}\nu \;N_{}^{2}\exp\left[-2l\frac{F}{kT}\right]\exp\left[\frac{S_{f}+S_{m}}{k}\right]\exp\left[-\frac{H_{A}^{0}-\sum_{i=0}^{l}E_{V}^{i}+lF}{kT}\right].\;$$

According to Equation (6), the change in the formation probability of a vacancy with charge *j*(*l*), with respect to the neutral one, is determined by the factor $\Delta_{-j}=j(2E_{cb}-3F)+\sum_{i=1}^{j}E_{V}^{i}$ for the negatively charged vacancy and the factor $\Delta_{+l}=3lF-\sum_{i=1}^{l}E_{V}^{i}$ for the positively charged one. Both factors are the functions of Fermi level position that depends on the carrier concentration. Under the ∆*_−j_* > 0 (∆*_+l_* > 0) condition, the neutral vacancy formation probability will exceed that for the negatively (positively) charged ones, and vice versa for ∆*_−j_* < 0 (∆*_+l_* < 0).

**Figure 6:**
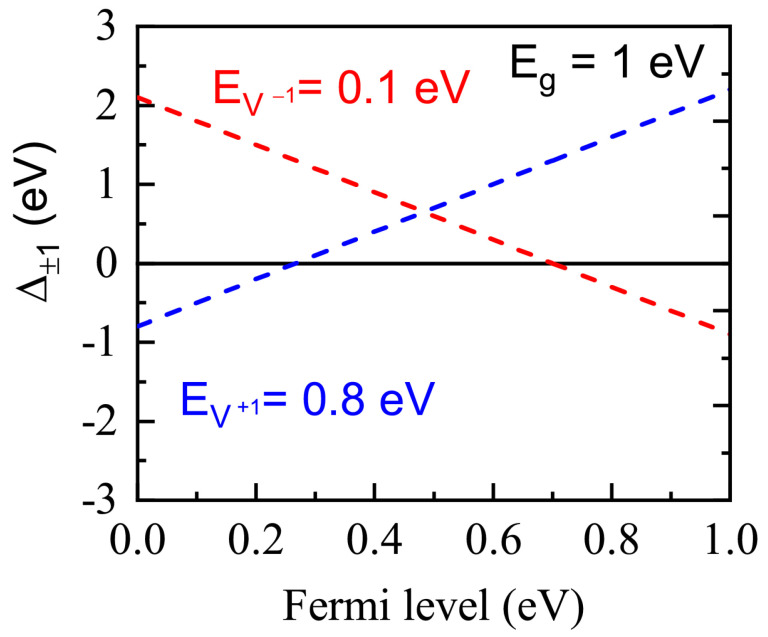
The ∆*_−j_* (red) and ∆+*_l_* (blue) values calculated as functions of the Fermi level position (0 corresponding to *F* = *E_vb_* and 1 to *F* = *E_cb_*, respectively) for a model material with *E_g_* = 1 eV and single-charged vacancies with the electron ionization energies of $E_{V}^{-1}$ = 0.1 eV and $E_{V}^{+1}$ = 0.8 eV.

Factors ∆*_−j_* and ∆*_+l_*, calculated for a model material with *E_g_* = 1 eV and single-charged vacancies with the ionization energies of $E_{V}^{-1}$ = 0.1 eV and $E_{V}^{+1}$ = 0.8 eV as the function of Fermi level position, are shown in Figure 6. The Fermi energy appropriated to ∆*_−_*_1_ < 0 should be shifted into the conduction band direction and to ∆*_+_*_1_ < 0 into the valence band direction.

Since various charge states are possible for a vacancy at a generation, the vacancy can be recharged after the formation. The kinetics of the neutral ($N_{VC}^{0}$) and single negatively charged ($N_{VC}^{-1}$) cationic vacancy concentrations, as well as neutral ($N_{VA}^{0}$) and single-positively charged ($N_{VA}^{+1}$) anionic vacancy concentrations, are described by the systems of kinetic equations that are presented in Appendix A.

Let us demonstrate the vacancies concentration dynamics in the bulk AlAs crystal. It is known that, for III-V compounds, the vacancy is found to exist in 0, −1, −2, and −3 charge states in the cationic sublattice and in 0, +1, +2, and +3 charge states in the anionic sublattice [52]. However, even for well-studied GaAs, the vacancy formation and migration enthalpies are still the subject of debates [24,29,53,54,55,56,57,58,59,60]. A fortiori, the vacancy formation parameters and their ionization energy in the different charged states for AlAs, are not well-established data. Therefore, one cannot quantitatively describe the vacancy formation dynamics. Here we will take into account neutral and single charged vacancies only, and that approximation is quite enough to qualitatively demonstrate the charge-carriers effect on the vacancy creation. Some vacancy parameters for AlAs (proposed in the literature) are presented in Table 1. Since the configuration entropy has a dominant contribution in $S_{f}^{}$ and $S_{m}^{}$ parameter values [28], in our estimated calculation, we used the formation and migration entropy values determined for GaAs, which is the crystal with a lattice similar to that for AlAs [55,59]. In general, vacancy migration enthalpies differ in different sublattices. However, for simplicity, we can assume that $H_{m}^{0}(V_{Al})\approx$$H_{m}^{0}(V_{As})$. We use here $H_{m}^{0}$ = 2.72 eV determined for AlGaAs [61]. The AlAs Debye frequency was determined, following [62], as $\nu =(TD_{AlAs}/TD_{GaAs}^{})\cdot \nu_{GaAs}$, where TD_AlAs_ and TD_GaAs_ are AlAs and GaAs Debye temperatures, respectively, and ν_GaAs_ is the GaAs Debye frequency [28]. The vacancy ionization levels taken from [63,64] are the same as for GaAs.

The effect of charged vacancy creation on the Fermi energy that reflects the change in the charge-carrier concentration in a crystal was calculated with the used neutrality equation.

**Figure 7:**
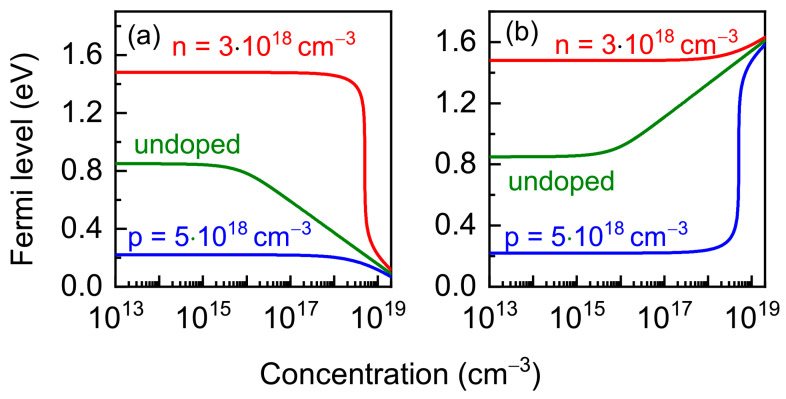
Fermi level energy calculated in AlAs as a function of the negatively V_Al_ (**a**) and positively V_As_ (**b**) charged vacancy concentration for temperature 1100 K (*E*_g_ = 1.822 eV). Cases: *N*_A_ << *N*_D_ = 3 × 10^18^ cm^−2^ (red), undoped (olive) and *N*_D_ << *N*_A_ = 5 × 10^18^ cm^−2^ (blue).

The calculation results are presented in Figure 7. The Fermi energy position is determined by the doping type and level. When the charged vacancy concentration exceeds the free charge carrier concentration, the Fermi energy is controlled by the concentration of charged vacancy.

**Figure 8:**
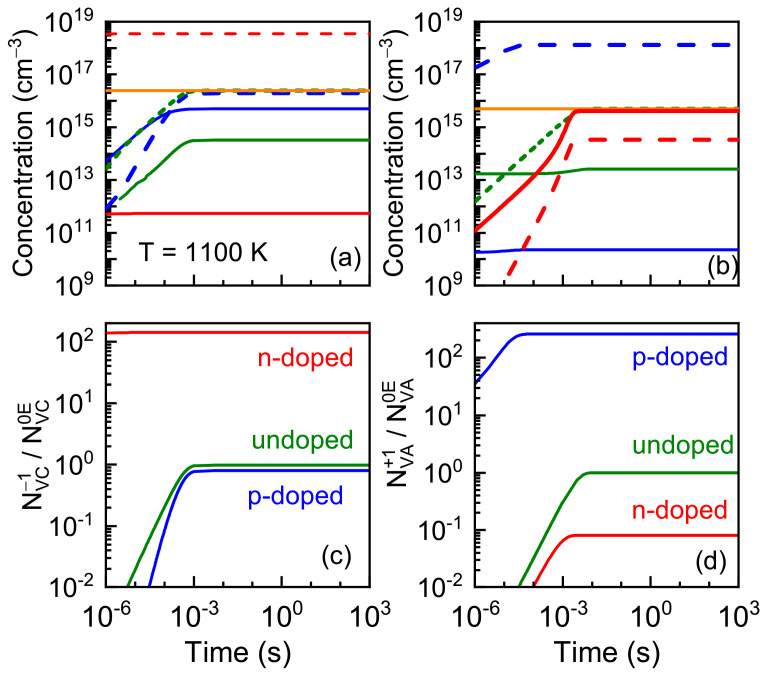
Dynamics of the neutral (solid lines) and single-charged (dashed lines) vacancy concentration (**a**,**b**), as well as the ratio of the charged vacancy to equilibrium neutral vacancy concentrations $N_{V}^{0E}$ (**c**,**d**), calculated at different doping levels (red—*n*-doped (with donor concentration 3 × 10^18^ cm^−3^), blue—*p*-doped (with acceptor concentration 5 × 10^18^ cm^−3^), olive—undoped) at *T* = 1100 K for: (**a**,**c**) the cationic AlAs sublattice, (**b**,**d**) the anionic AlAs sublattice.

The dynamics of neutral and charged vacancy concentrations, as well as the ratio of charged vacancy to equilibrium neutral vacancy concentrations ($N_{V}^{0E}$), were calculated in cationic (Figure 8a,c) and anionic (Figure 8b,d) AlAs sublattices for different doping levels. The vacancy concentration rises with saturation. For cationic sublattices, the charged vacancy concentration exceeds the neutral ones in all cases. However, the saturation level for the charged *V*_Al_ vacancy concentration in undoped and *p*-doped AlAs corresponds to the equilibrium neutral vacancies concentration which shown by orange line (see Figure 8a). In undoped and *p*-doped cases, the factor ∆_−1_ that reflected the formation probability of charged vacancy, with respect to the neutral one, in the cationic sublattice is positive. Therefore, the vacancies appear mainly as a result of the neutral Frenkel pair formation and those capturing electrons after the pair separation. On the other hand, in the *n-*doped material, the charged vacancy concentration *V*_Al_ exceeds strongly the equilibrium neutral vacancies concentration, and that corresponds to the case of negative factor ∆_−1_.

In the anionic sublattice, the charged vacancy concentration *V*_As_ strongly exceeds the equilibrium neutral vacancy concentration (shown by orange line) in the *p-*doped material (∆_+1_ < 0), while, in the undoped AlAs, the saturation level for the charged *V*_As_ vacancy concentration corresponds to the equilibrium neutral vacancies concentration, and in the case of the *n-*doped material, neutral vacancies dominate over charged ones, as it is shown in Figure 8b, and that corresponds to ∆_+1_ > 0. In both of these, cases the neutral vacancy concentration does not reach the equilibrium concentration, and that indicates that charging of the vacancies is produced as a result of the neutral pair generation.

Since vacancies control interdiffusion in III–V heterostructures [65,66], the diffusivity factor for substitution impurities (*D*) is proportional to the vacancy concentration [41]. Therefore, we take into account that indium (antimony) diffusion $D=D_{V}N_{V}^{}/N$ in the (In,Al)As/AlAs (Al(Sb,As)/AlAs) heterostructure can be described by the equation:

(7a) $$\frac{\partial C_{In}(z,t)}{\partial t}=\frac{D_{VC}}{N}\frac{\partial }{\partial z}\left[\left(N_{VC}^{0}(z,t)+N_{VC}^{-1}(z,t))\frac{\partial }{\partial z}C_{In}(z,t\right)\right],$$

(7b) $$\frac{\partial C_{Sb}(z,t)}{\partial t}=\frac{D_{VA}}{N}\frac{\partial }{\partial z}\left[\left(N_{VA}^{0}(z,t)+N_{VA}^{+1}(z,t))\frac{\partial }{\partial z}C_{Sb}(z,t\right)\right].$$

The change in the generation rate and, as a consequence, the charged vacancy concentration in bulk AlAs with a change in the doping type and level (which determines the Fermi level position) provides a qualitative explanation of our experimental results (the acceleration of In diffusion in *n*-doped structures and the acceleration of Sb diffusion in *p*-doped structures). However, it is necessary to consider one more feature of heterostructures revealed in [16], which, as shown in the next subsection, can have a noticeable effect on the spatial distribution of the charged vacancy creation rate.

### 4.2. Spatial Distribution of Vacancy Generation Rate in Heterostructures

**Figure 9:**
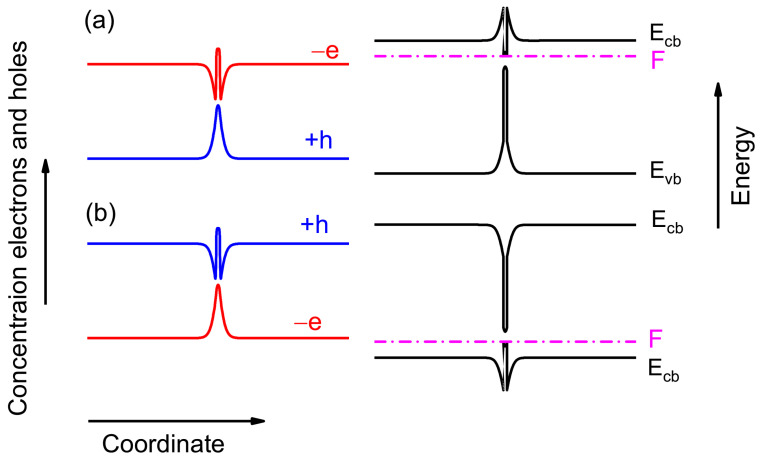
Schematic band diagrams and spatial electron −e and hole +h distributions. (**a**) *n*-doped heterostructure (**b**) *p*-doped heterostructure.

The fundamental difference between a bulk semiconductor and a heterostructure with QWs and QDs is the spatial distribution of electrons and holes [16]. A bulk semiconductor (except near a surface region) has a uniform equilibrium charge carrier distribution. Therefore, the spatial distribution of the charged vacancy generation rate in the bulk material is also uniform. The situation is changed drastically in heterostructures with QWs and QDs. The charge carriers are captured from the matrix in a region with a smaller bandgap, and that results in the nonuniform spatial concentration of electrons and holes in the heterostructure, as is shown in Figure 9. The nonuniform carrier distribution results in the band bending, as one can see in these figures. Therefore, the vacancy creation rate becomes spatially uneven. As has been shown recently in [16], the local generation rate of a charged vacancy changing in the region around QW is due to the band bending. 

The dynamics of the neutral and single negatively (positively) charged vacancy concentration in the heterostructure is described by the equation systems that are presented in Appendix B.

### 4.3. Band Diagrams of Heterostructures with QDs and Carrier Distribution

Let us look at the energy diagram calculated at high temperatures for (In,Al)As/AlAs and Al(Sb,As)/AlAs heterostructures with QDs in order to demonstrate the spatially uneven charge-carrier distribution. The energy spectra of heterostructures and charge distribution were calculated using the NEXTNANO3 nanodevice simulation tool [67]. Lattice constants, effective masses of electrons and holes in AlAs, InAlAs, and AlSbAs alloys, valence band offset, and its temperature dependences were taken from [68]. The calculations took into account the deformation, deformation potentials, and generation and distribution of charge carriers. The calculation methods are shown in [34,69,70,71]. For simplicity, the exciton correction for the energy levels was neglected.

**Figure 10:**
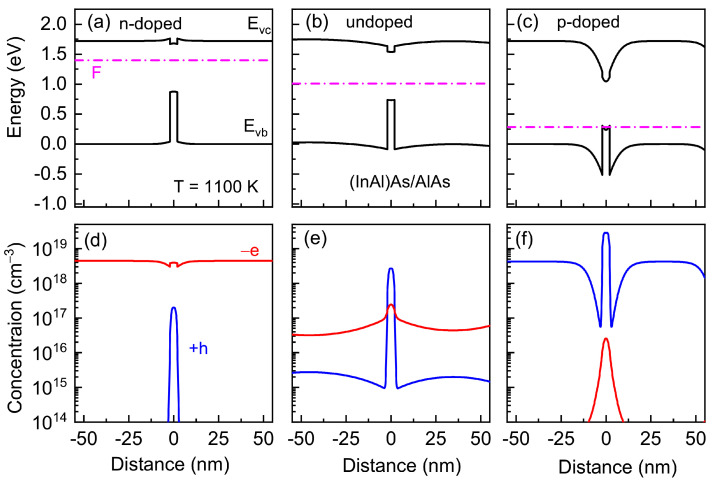
Band diagrams (**a**–**c**) and spatial electron −e (red) and hole +h (blue) distributions (**d**–**f**) calculated for *n*-doped (**a**,**d**), undoped (**b**,**e**), and *p*-doped (**c**,**f**) heterostructures with (In,Al)As/AlAs QDs.

**Figure 11:**
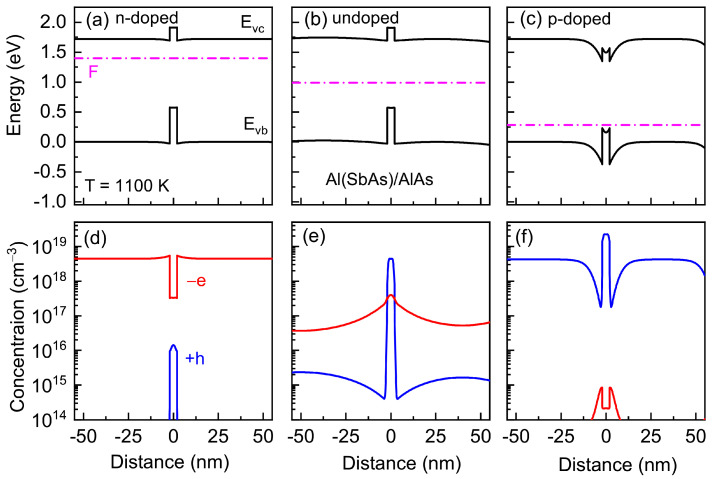
Band diagrams (**a**–**c**) and spatial electron −e (red) and hole +h (blue) distributions (**d**–**f**) calculated for *n*-doped (**a**,**d**), undoped (**b**,**e**), and *p*-doped (**c**,**f**) heterostructures with Al(Sb,As)/GaAs QDs.

The energy diagrams and charge-carrier distribution calculated for *n*-doped, undoped, and *p*-doped (In,Al)As/AlAs and Al(Sb,As)/AlAs heterostructures are shown in Figure 10 and Figure 11, respectively. A specific feature of both heterostructures is that the localizing potential for electrons in the conduction band is noticeably smaller than that for holes in the valence band.

**Figure 12:**
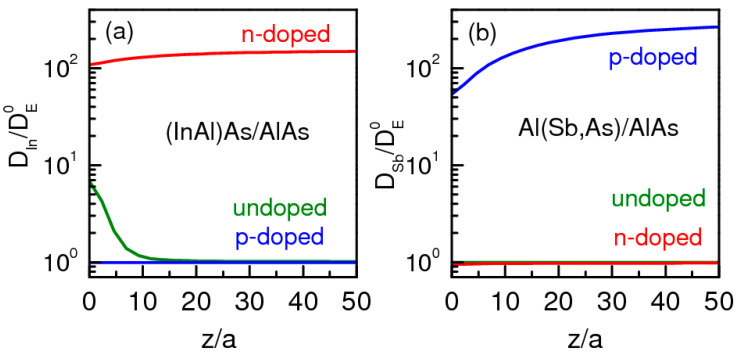
Diffusivity factor distribution calculated for (**a**) indium in (In,Al)As/AlAs and (**b**) antimony in Al(Sb,As)/AlAs heterostructures: *n*-doped (red), undoped (olive), and *p*-doped (blue). The diffusivity factors are normalized to the value $D_{E}^{0}$ determined for the neutral vacancies’ equilibrium concentration.

The low electron capacity in the QD region leads to the spatial electron distribution in *n*-doped heterostructures, which is very similar to that in bulk AlAs. As a result, in the *n*-doped heterostructures with (In,Al)As/AlAs QDs, the indium diffusivity factor, that is determined by the negatively charged Al vacancy distribution, is practically spatially uniform as in a bulk AlAs crystal (only a small dispersion is observed near the QD/matrix interface due to a small band-bending, as it is shown in Figure 12a). However, in the p-doped heterostructures, the charge distribution is quite different from that in the n-doped ones. The collection of positively charged holes in QDs leads to a strong spatially nonuniform charge distribution. As a result, the antimony diffusivity factor, that is determined in p-doped heterostructures with Al(Sb,As)/AlAs QDs by the positively charged As vacancy distribution, becomes spatially nonuniform (it strongly decreases near the QD/matrix interface, as is shown in Figure 12b). Note that the charge distribution in *p*-doped (*n*-doped) heterostructures does not affect the vacancy concentration and diffusivity factor distributions in (In,Al)As/AlAs QDs (Al(Sb,As)/AlAs QDs) since the neutral vacancies with the spatially uniform distribution are dominant.

The concentration of electrons and holes integrated over all layers in undoped heterostructures coincides due to the electrical neutrality condition. However, one can see an unbalance of electrons and holes in the QD region (see Figure 10e and Figure 11e) arising, as we have shown recently in [16], as a result of the difference in the charge carrier generation rate in the wide-gap matrix and narrow-gap quantum dot material and a subsequent redistribution of these carriers in the heterostructure.

In the framework of our simple model, we calculated the diffusivity factor spatial distributions for p-doped (with acceptor concentration 5 × 10^18^cm^−3^), undoped, and n-doped (with donor concentration 3 × 10^18^cm^−3^) heterostructures with (In,Al)As/AlAs and Al(Sb,As)/AlAs QDs, taking into account the spatial electron and hole distributions. The calculated results are shown in Figure 12. The calculated diffusivity factors are normalized to the spatially uniformed diffusivity factor values determined for the neutral vacancies’ equilibrium concentration. One can see that increasing the electron density in the QD/matrix heterojunction region increases the charged vacancy generation rate and, as a result, the indium diffusivity factor in the (In,Al)As/AlAs heterostructure. Therefore, a noticeable mixing of materials takes place even in the undoped heterostructure, as can be seen from the PL band blue-shift in Figure 4a. On the other hand, decreasing the hole density in the QD/matrix heterojunction region in the Al(Sb,As)/AlAs heterostructure results in the domination for the neutral vacancy formation that does not increase the antimony diffusivity factor. Therefore, the mixing of materials is practically the same for undoped and n-doped heterostructures, as can be seen in Figure 4b; in both cases, the PL band blue-shift is negligible. Thus, one can see that the calculations in the framework of the model give a good explanation for all features of our experimental data. 

Note that, including the dynamics of complex defects (such as bi-vacancies or vacancy-point defect complexes), formation in the model will lead to insignificant quantitative changes in the calculated diffusion parameters for low vacancy concentrations. In this case, there will be a slight decrease in the diffusion coefficient, since some vacancies are excluded from the diffusion process. On the other hand, the formation of complex defects slightly affects the processes associated with the nonuniform spatial distribution of charge-carriers. However, at high vacancy concentrations, complex defect formation can greatly change the diffusion pattern.

## 5. Conclusions

The effect of the charge carrier concentration on the high temperature vacancy-mediated intermixing in AlAs-based heterostructures with QDs formed in the cationic and anionic sublattices was studied. The intermixing degree in the cationic (anionic) sublattice is accelerated with the increases in the electron (hole) concentration as a result of an increase in the negatively (positively) charged vacancy formation rate. We demonstrated that the diffusion coefficient in such heterostructures can be nonuniform, and that is due to the nonuniformity for the charged vacancy creation that stems from a gradient of the carrier concentration in the QD/matrix heterointerface region. Space charge regions arise in the heterostructures due to the carriers capture in the QDs from the doped matrix. A theoretical model of the vacancy-mediated high-temperature diffusion in doped semiconductor heterostructures was developed. The features of experimental data are well explained in the framework of this model.

## Figures and Tables

**Table 1:** Vacancy parameters in AlAs used in calculation: formation enthalpy $H_{f}^{0}$ and entropy $S_{f}^{}$, migration enthalpy $H_{m}^{0}$ and entropy $S_{m}^{}$, ionization energy in different charged states $E_{V}^{-1}$ /$E_{V}^{+1}$, and ν is the AlAs Debye frequency.

Parameter	Al Vacancy Value	As Vacancy Value
$H_{f}^{0}$ (eV)	3.62 [24]	3.83 [24]
$H_{m}^{0}$ (eV)	2.72 [61]	
$S_{f}^{}$	7.3 × *k* [59]	
$S_{m}^{}$	11.3 × *k* [55]	
*ν* (Hz)	1.3 × 10^13^	
$E_{V}^{-1}$/$E_{V}^{+1}$ (eV)	*E_vb_* + 0.11 [64]	*E_cb_* − 0.14 [63]

## Data Availability

Data are available from the authors on request.

## Appendix A

The kinetics of the neutral ($N_{VC}^{0}$) and single negatively charged ($N_{VC}^{-1}$) vacancy concentration in the cationic sublattice is described by the system of kinetic equations:

(A1) $$\begin{array}{c} N_{IC}^{}(t)=N_{VC}^{0}(t)+N_{VC}^{-1}(t),\\ N_{ce}\exp\left[\frac{F(t)-E_{cb}^{}}{kT}\right]-N_{vh}\exp\left[-\frac{F(t)}{kT}\right]+N_{VC}^{-1}(t)-N_{D}+N_{A}=0, \end{array}$$

where $G_{C}^{0}$ and $R_{C}^{0}$ are neutral vacancy creation and recombination probabilities in the cationic sublattice, and $\gamma^{-}$ is a trapping coefficient of an electron for a neutral vacancy. $N_{D}^{}$ and $N_{A}^{}$ are shallow donor and acceptor concentrations (at high temperatures, all impurities are assumed to be ionized). Note that, here and in what follows, we examine a low compensated semiconductor with *N_A_* << *N_D_* or *N_D_* << *N_A_*.

In the case of the neutral ($N_{VA}^{0}$) and single positively charged ($N_{VA}^{+1}$) vacancies in the anionic sublattice, the system of kinetic equations is:

(A2) $$\begin{array}{c} \frac{\partial N_{VA}^{0}(t)}{\partial t}=N^{2}G_{A}^{0}-N_{VA}^{0}(t)N_{IA}^{}(t)R_{A}^{0}-\gamma^{+}N_{VA}^{0}(t)+\gamma^{+}N_{VA}^{+1}(t)\exp\left[-\frac{E_{V}^{+1}-F(t)}{kT}\right],\\ \frac{\partial N_{VA}^{+1}(t)}{\partial t}=N^{2}G_{A}^{0}\exp\left[\frac{E_{V}^{+1}-3F(t)}{kT}\right]-N_{VA}^{+1}(t)N_{IA}^{}(t)R_{A}^{0}+\gamma^{+}N_{VA}^{0}(t)-\gamma^{+}N_{VA}^{+}(t)\exp\left[-\frac{E_{V}^{+1}-F(t)}{kT}\right],\\ N_{IA}^{}(t)=N_{VA}^{0}(t)+N_{VA}^{+1}(t),\\ N_{ec}\exp\left[\frac{F(t)-E_{cb}^{}}{kT}\right]-N_{vh}\exp\left[-\frac{F(t)}{kT}\right]-N_{VA}^{+1}(t)-N_{D}+N_{A}=0 \end{array}$$

where $G_{A}^{0}$ and $R_{A}^{0}$ are neutral vacancy generation and recombination probability in the anionic sublattice, and $\gamma^{+}$ is a coefficient of an electron emission from a neutral vacancy.

## Appendix B

The dynamics of the neutral and single negatively charged vacancy concentrations in the heterostructure can be described as:

(A3) $$\begin{array}{c} \frac{\partial N_{VC}^{0}(z,t)}{\partial t}=N^{2}G_{C}^{0}-N_{VC}^{0}(z,t)N_{IC}^{}(z,t)R_{C}^{0}-\gamma^{-}N_{VC}^{0}(z,t)+\gamma^{-}N_{VC}^{-1}(z,t)\exp\left[\frac{F(t)-E_{V}^{-1}(z,t)}{kT}\right]+D_{VC}\left[\frac{\partial^{2}N_{VC}^{0}(z,t)}{\partial z^{2}}\right],\\ \frac{\partial N_{VC}^{-1}(z,t)}{\partial t}=N^{2}G_{C}^{0}\exp\left[-\frac{E_{V}^{-1}(z,t)+2E_{cb}(z,t)-3F(t)}{kT}\right]+\gamma^{-}N_{VC}^{0}(z,t)-\\ \gamma^{-}N_{VC}^{-1}(z,t)\exp\left[-\frac{F(t)-E_{V}^{-1}(z,t)}{kT}\right]-N_{VC}^{-1}(z,t)N_{IC}^{}(t)R_{C}^{0}+D_{V}\left[\frac{\partial^{2}N_{VC}^{-1}(z,t)}{\partial z^{2}}\right],\\ N_{ec}\exp\left[\frac{F(t)-E_{cb}^{}(z,t)}{kT}\right]-N_{vh}\exp\left[-\frac{F(t)}{kT}\right]+N_{VC}^{-1}(z,t)-N_{D}+N_{A}=0,\\ \frac{\partial C_{In}^{}(z,t)}{\partial t}=\frac{D_{VC}}{N}\frac{\partial }{\partial z}\left[\left(N_{VC}^{0}(z,t)+N_{VC}^{-1}(z,t))\frac{\partial }{\partial z}C_{In}(z,t\right)\right], \end{array}$$

and, for the case of neutral and single positively charged vacancies, as:

(A4) $$\begin{array}{c} \frac{\partial N_{VA}^{0}(z,t)}{\partial t}=N^{2}G_{A}^{0}-N_{VA}^{0}(z,t)N_{IC}^{}(z,t)R_{A}^{0}+\gamma^{+}N_{VA}^{+1}(z,t)-\gamma^{+}N_{VA}^{0}(z,t)\exp\left[\frac{F(t)-E_{V}^{+1}(z,t)}{kT}\right]+D_{VA}\left[\frac{\partial^{2}N_{VA}^{0}(z,t)}{\partial z^{2}}\right],\\ \frac{\partial N_{VA}^{+1}(z,t)}{\partial t}=N^{2}G_{A}^{0}\exp\left[-\frac{3F(t)-E_{V}^{+1}(z,t)}{kT}\right]-\gamma^{+}N_{VA}^{+1}(z,t)+\gamma^{+}N_{VC}^{0}(z,t)\exp\left[\frac{F(t)-E_{V}^{+1}(z,t)}{kT}\right]-\\ N_{VA}^{+1}(z,t)N_{IA}^{}(t)R_{A}^{0}+D_{VA}\left[\frac{\partial^{2}N_{VA}^{+1}(z,t)}{\partial z^{2}}\right],\\ N_{ec}\exp\left[\frac{F(t)-E_{cb}^{}(z,t)}{kT}\right]-N_{vh}\exp\left[-\frac{F(t)}{kT}\right]-N_{VA}^{+1}(z,t)-N_{D}+N_{A}=0,\\ \frac{\partial C_{Sb}^{}(z,t)}{\partial t}=\frac{D_{VA}}{N}\frac{\partial }{\partial z}\left[\left(N_{VA}^{0}(z,t)+N_{VA}^{+1}(z,t))\frac{\partial }{\partial z}C_{Sb}(z,t\right)\right]. \end{array}$$
